# Supplementary material for: ‘Through the drawings…they are able to tell you straight’: Using arts-based methods in violence research in South Africa
Source: PLOS Glob Public Health. 2023 Oct 9;3(10):e0002209. doi: 10.1371/journal.pgph.0002209 (PMC10561840; doi:10.1371/journal.pgph.0002209)
Supplement: S1 File — (DOCX) [file pgph.0002209.s001.docx]

Feeling Faces Game

The aim of this ‘game’ is to help participants understand the basic feelings of happy, sad, scared and angry in the form of faces.  Understanding these feelings is comprised of being able to name them, recognise them for oneself and recognize them in others.  It provides a common understanding regarding some of the measures in the questionnaire that incorporate feeling words. The game is also intended to be fun and playful and help with engaging child participants in the research.

1. The interviewer draws 4 circles on the laminated sheet using the markers provided.
2. The interviewer fills in the 4 circles with eyes, mouth, eyebrows that depict ‘happy’, ‘sad’, ‘scared’, and ‘angry’.
3. The interviewer points to the face and asks the child what feeling it represents.  If the child gets it correct, give high praise.  If the child gets it wrong, name the feeling and write the word of the feeling next to the face.  Do this with each of the faces on the laminated sheet. Ensure that feeling names are written next to feeling faces.
4. Then engage in a charades game with the child (the laminated sheet should be placed between interviewer and child). The interviewer chooses a feeling in their mind and acts/shows it on their face and asks the child to guess their feeling.  Do this for all four feelings and see if the child is able to guess the interviewer’s face.  Make this fun and engaging with high praise if the child guesses correctly. If they are unable to, ask the child to point to the face on the laminate sheet that best shows the interviewer’s face.  Help the child understand the interviewer’s feeling face by showing how the eyes look and how the mouth looks etc.  The idea is to ‘learn’ the feeling.
5. Once the interviewer is done with all four feelings, invite the child to have a turn.
6. Ask the child to look at the laminated sheet and choose one of the four feelings to show on their face for the interviewer to guess.
7. Continue until the child has ‘enacted’ all four of their feelings.
8. End with encouragement for doing a good job in the feelings game.
