# Supplementary material for: ‘Through the drawings…they are able to tell you straight’: Using arts-based methods in violence research in South Africa
Source: PLOS Glob Public Health. 2023 Oct 9;3(10):e0002209. doi: 10.1371/journal.pgph.0002209 (PMC10561840; doi:10.1371/journal.pgph.0002209)
Supplement: S2 File — (DOCX) [file pgph.0002209.s002.docx]

**House-and-community plan - (1) Drawing, (2) Clay figures, (3) “Zoom” in**

**Sources**: Fouché & Joubert (2000); Fouché (2006)

The technique's goal is to focus on the child’s daily movements from one place to another.  With this visual and interactive play-related communication technique, it is possible to identify places where a child feels secure and identify areas and situations where they feel threatened.  Through this technique, a child is also allowed to express both positive and negative emotions.

1. **Drawing**
2. The interviewer draws with the child’s assistance, a plan of his/her home (always ask the child if he/she has more than one home and make a note to draw that house as well)
3. **Clay figures**
4. Next, make clay models representing family members.  The child or the interviewer can write the people’s names next to their clay figures / furniture can also be drawn in.
5. Optional: Also draw the area surrounding the house and the neighbouring houses (e.g. house shops, shebeens, open fields, schools, etc.).
6. Ask the child if he/she wants to change anything
7. Optional: The interviewer/child can also make clay models representing the neighbours
8. **“Zoom” in**

Questions like the following are asked to explore further by “zooming” into the family life.

| Sleeping arrangements | |
| --- | --- |
| “Who sleeps where.”  “Put the clay figures in the beds/spaces where they are sleeping.”  “Is there anyone else who is sometimes sleeping over your house? Where? | Use clay figures and move around. |
| Caretaking | |
| “Who wakes you up in the morning? How does mom/dad wake you up?”  “What does mom/dad do when you don’t want to get up?”  “What do you do after you got up?  “Who prepares food…   “What food do you eat in morning/ afternoon/evening?  What happens if there is no bread/tea/milk/pap”?  “Who dresses you/baths you….”?  “What do you do when you get scared/feel sick?”  “How do you get to school…  “What do you take in your lunch box…what will happen if you forget your lunchbox at home…?”  “How do you get from school?  “Where do you go after school?”  “Who is looking after you?”  “What do you like/dislike of being there?”   “Who puts you to bed?”  Anyone else/visitors/sleep over/ | Move clay figures around |
| Boundaries | |
| “Where do you bathe/go to the toilet??  “What does mom/dad/other people staying in the house/community do when you are in the toilet, and they want to come in quickly?  Who baths you/wipe your bum?”  “What do you see when dad/mom/brother comes into the toilet/bathroom/uses the toilet?” | Add to drawing and indicate with clay figures |
| Conflict / violence | |
| “What happens if mom/dad/uncle gets angry?”  “Who gets angry the most in your house? And then what, how etc. “  “What happens if mom/dad is mad at you / at one another? “ | Indicate with clay figures |

**Safe and unsafe - (1) Explain, (2) Clarify, (3) Explore & Follow-up**

1. **Explain** the meaning of safe and unsafe

- **Unsafe:**  When you feel uneasy, when you are scared when you want to get away. It is like the RED traffic light –TELLS US TO  STOP OTHERWISE WE MAY GET HURT
- **SAFE**: When you are not scared, you feel at ease; you like to be with that person. Like the GREEN traffic light –TELLS US IT IS SAFE TO GO – WE MAY NOT GET HURT

1. **Clarify** whether the child understands the meaning of safe and unsafe using neutral topics and red and green colouring pencils/crayons

**Test 1:** Let’s say someone at school or where you play outside is very rude to you, and it makes you feel sad – do you feel safe (show the green pencil) or unsafe (show the red pencil).

**Test 2**: Let’s say someone at school or where you play outside is nice to you, you are not scared of them, do you feel safe (show the green pencil) or unsafe (show the red pencil).

Do the above for two rounds with different topics. If the child gets it right twice – move on. If the child only gets it right once – do another; if the child gets it right the second time move on. Suppose the child does not get it right twice. Do no go to the next step as the child’s responses may not be accurate.

1. **Explore & Follow-up :**
2. The child is then requested to identify and mark all the safe havens with a green or red pen (make a cross or tick)
3. And the unsafe places with a red pen.
4. After the child has marked the safe /unsafe havens, explore the **safe** places/persons

- “Let me know if I'm wrong – (now pointing to the green ticks/ crosses) - you feel safe here, here and here. Is it still ok? Do you want to change anything? Ok.”
- “What does mommy do to make you feel safe?” Etc.

1. Explore and follow up on the unsafe places/persons.

- “I see you feel unsafe in your room:  Can you tell me what happened/what makes it unsafe?”
- “I see that you don’t like uncle Thabo/James. So tell me, what about him/what does he do that you don’t like?”

**References**

Fouché. A., & Joubert, J.M.C. (2003). Play techniques in the assessment of sexually abused black children. *Acta Criminologica, 16*, 12 - 20.

Fouché, A. (2006). Assessment of the Sexually Abused Child. In Spies, G.M. (Ed.), *Sexual abuse: dynamics, assessment and healing*. Pretoria: Van Schaik Publishers.
