# Supplementary material for: ‘Through the drawings…they are able to tell you straight’: Using arts-based methods in violence research in South Africa
Source: PLOS Glob Public Health. 2023 Oct 9;3(10):e0002209. doi: 10.1371/journal.pgph.0002209 (PMC10561840; doi:10.1371/journal.pgph.0002209)
Supplement: S3 File — (PDF) [file pgph.0002209.s003.pdf]

UNIVERSITY OF THE WITWATERSRAND, JOHANNESBURG	HUMAN RESEARCH ETHICS COMMITTEE (MEDICAL)

Office of the Deputy Vice-Chancellor (Research and Innovation)

TO: Dr F Meinck, Professor N Christofides, et al
School of Public Health
Medical School
University

E-mail: Nicola.Christofides@wits.ac.za

CC: Supervisor: Not applicable
<>
and <HREC-Medical Research Office@wits.ac.za>

FROM: Mr Iain Burns
Human Research Ethics Committee (Medical)
Tel: 011 717 1252

E-mail: Iain.Burns@wits.ac.za

DATE: 2021/07/08

REF: R14/49

PROTOCOL NO: **M190949** (This is your ethics application reference number. Please quote it in all enquiries, oral or written, relating to this study.)

PROJECT TITLE: *Interrupting the intergenerational transmission of violence: a mixed-methods longitudinal study in South Africa*

Please find attached the Clearance Certificate for the above project. I hope it goes well and that an article in a recognized publication comes out of it. This will reflect well on your professional standing and contribute to Government funding of the University.

MSWorks2000/Iain0007/Clearscan.wps

UNIVERSITY OF THE
WITWATERSRAND,
JOHANNESBURG

R49 Dr F Meinck, Professor N Christofides, et al

HUMAN RESEARCH ETHICS COMMITTEE (MEDICAL)
CLEARANCE CERTIFICATE NO. M190949

NAME:
(Principal Investigator) Dr F Meinck, Professor N Christofides, et al

DEPARTMENT: School of Public Health
Medical School
University

PROJECT TITLE: *Interrupting the intergenerational transmission of violence: a mixed-methods longitudinal study in South Africa*

DATE CONSIDERED: 2019/09/27

DECISION: Approved unconditionally

CONDITIONS: This approval applies to the pilot study only
A subsequent discussion with the HREC (Med) will inform further developments

SUPERVISOR: Not applicable

APPROVED BY: [signature]
Dr CB Penny, Chairperson, HREC (Medical)

DATE OF APPROVAL: 2021/07/08

This Clearance Certificate is valid for 5 years from the date of approval. An extension may be applied for.

DECLARATION OF INVESTIGATORS

To be completed in duplicate and **ONE COPY** returned to the Research Office secretariat on the 3rd floor, Phillip Tobias Building, Parktown, University of the Witwatersrand, Johannesburg.

I/we fully understand the conditions under which I am/we are authorized to carry out the above-mentioned research and I/we undertake to ensure compliance with these conditions. Should any departure be contemplated from the research protocol as approved, I/we undertake to submit details to the Committee. **I agree to submit a yearly progress report**. When a funder requires annual re-certification, the application date will be one year after the date when the study was initially reviewed. In this case, the study was initially reviewed in **September** and therefore reports and re-certification will be due in the month of **September** each year. Unreported changes to the study may invalidate the clearance given by the HREC (Medical).

______________________________ ______________________
Signature of Principal Investigator Date

SCHOOL *of* SOCIAL *and* POLITICAL SCIENCE
Social Policy

The University of Edinburgh
Chrystal Macmillan Building
15A George Square
Edinburgh EH8 9LD

Telephone: 0131 651 5471
Email: sudeepa.abeysinghe@ed.ac.uk

Dr Franziska Meinck
School of Social and Political Science
Social Work Subject Area

20/02/20

Research Ethics Approval

Ref No: 264227

INTERRUPT_VIOLENCE (Interrupting the Intergenerational Transmission of Violence: a longitudinal mixed-methods study in South Africa)

Dear Franziska,

The above application has been considered on behalf of the School of Social and Political Science Research Ethics review process, in accordance with the procedures laid down by the University for ethical approval of all research involving human participants.

I am pleased to inform you that, on the basis of the information provided to the Ethics Committee, the proposed research has been judged as meeting appropriate ethical standards, and accordingly approval has been granted at level 3.

Should there be any subsequent changes to the stated protocol, or if any ethical challenges unexpectedly arise in the field, you should submit details to the Ethics Committee for consideration. Given the complexity of this project, the committee also recommends a post-project review, and you should get in touch to trigger that process when necessary.

Yours sincerely,

Dr. Sudeepa Abeysinghe
Deputy Director of Research (Ethics and Integrity)

health
MPUMALANGA PROVINCE
REPUBLIC OF SOUTH AFRICA

Indwe Building, Government Boulevard, Riverside Park, Ext. 2, Mbombela, 1200, Mpumalanga Province
Private Bag X11285, Mbombela, 1200, Mpumalanga Province
Tel l: +27 (13) 766 3429, Fax: +27 (13) 766 3458

Litiko Letemphilo | Departement van Gesondheid | UmNyango WezeMaphilo

Enq: 013 766 3766/3511
Ref: MP_202012_002

Research Approval Letter

Dr Franziska Meinck
VAL TRIANGLE CAMPUS
1174 Hendrick Van Eck Boulevard
Vanderbijlpark, 1900

TITLE: APPLICATION FOR RESEARCH APPROVAL: INTERRUPTING THE INTERGENERATIONAL TRANSMISSION OF VIOLENCE

Dear Dr Meinck

The Provincial Department of Health Research Committee has approved your research proposal in the latest format you sent.

- **Approval Reference Number:** **MP_202202_003**
- **Data Collection Period:** **20 December 2020 to 30 December 2022**
- **Approved Data Collection Facilities:** **Ehlanzeni District Municipality Offices**

Kindly ensure that conditions mentioned below are adhered to, and that the study is conducted with minimal disruption and impact on our staff, and also ensure that you provide us with a soft or hard copy of the report once your research project has been completed.

Conditions:
- *Researchers not allowed to make copies or take pictures of medical records.*
- *Kindly notify the facility manager a week BEFORE you start with data collection to ensure that conditions are conducive in the facility.*
- *The FINAL RESEARCH FINDINGS must be uploaded on the NHRD website.*

Kind regards

DR C NELSON
MPUMALANGA PHRC CHAIRPERSON
DATE: 28 | 02 | 2022

MPUMALANGA

Private Bag X1290, Potchefstroom
South Africa 2520

Tel: 086 016 9698
Web: http://www.nwu.ac.za/

North-West University Health Research Ethics Committee (NWU-HREC)

Tel: 018 299-1206
Email: Ethics-HRECApply@nwu.ac.za (for human studies)

07 July 2021

ETHICS APPROVAL LETTER OF STUDY

Based on approval by the North-West University Health Research Ethics Committee (NWU-HREC) on 07/07/2021, the NWU-HREC hereby approves your study as indicated below. This implies that the NWU-HREC grants its permission that, provided the general and specific conditions specified below are met and pending any other authorisation that may be necessary, the study may be initiated, using the ethics number below.

Study title: Interrupting the intergenerational transmission of violence: a mixed-methods longitudinal study in South Africa
Principal Investigator/Study Supervisor/Researcher: Dr F Meinck
Student: -

Ethics number:

N	W	U	-	0	0	3	2	9	-	2	0	-	A	1
Institution				Study Number						Year			Status	

Status: S = Submission; R = Re-Submission; P = Provisional Authorisation; A = Authorisation

Application Type: Single study
Commencement date: 07/07/2021
Expiry date: 31/07/2022

Risk:

Adults: Medium
Children: Category 3 – Greater than minimal risk with no prospect of direct benefit

Approval of the study is provided for a year, after which continuation of the study is dependent on receipt and review of a six-monthly monitoring report and the concomitant issuing of a letter of continuation. Monitoring reports are due at the end of July and February annually until completion.

General conditions:

While this ethics approval is subject to all declarations, undertakings and agreements incorporated and signed in the application form, the following general terms and conditions will apply:

- *The principal investigator/study supervisor/researcher must report in the prescribed format to the NWU-HREC:*
  - *six-monthly on the monitoring of the study, whereby a letter of continuation will be provided annually, and upon completion of the study; and*
  - *without any delay in case of any adverse event or incident (or any matter that interrupts sound ethical principles) during the course of the study.*
- *The approval applies strictly to the proposal as stipulated in the application form. Should any amendments to the proposal be deemed necessary during the course of the study, the principal investigator/study supervisor/researcher must apply for approval of these amendments at the NWU-HREC, prior to implementation. Should there be any deviations from the study proposal without the necessary approval of such amendments, the ethics approval is immediately and automatically forfeited.*
- *Annually a number of studies may be randomly selected for active monitoring.*
- *The date of approval indicates the first date that the study may be started.*
- *In the interest of ethical responsibility, the NWU-HREC reserves the right to:*
  - *request access to any information or data at any time during the course or after completion of the study;*

- *to ask further questions, seek additional information, require further modification or monitor the conduct of your research or the informed consent process;*
  - *withdraw or postpone approval if:*
    - *any unethical principles or practices of the study are revealed or suspected;*
    - *it becomes apparent that any relevant information was withheld from the NWU-HREC or that information has been false or misrepresented;*
    - *submission of the six-monthly monitoring report, the required amendments, or reporting of adverse events or incidents was not done in a timely manner and accurately; and/or*
    - *new institutional rules, national legislation or international conventions deem it necessary.*
- *NWU-HREC can be contacted for further information via Ethics-HRECApply@nwu.ac.za or 018 299 1206*

Special conditions of the research approval due to the COVID-19 pandemic:

Please note: Due to the nature of the study i.e. (face-to-face quantitative surveys and qualitative in-depth interviews with young people, their children and former primary caregivers will be conducted in a community setting), this study will be able to proceed during the current alert level, following receipt of the approval letter. No additional COVID-19 restrictions have been placed on the study, other than that which is indicated under the COVID-19 risk mitigation strategy as indicated in the application documentation. The researcher must, however, ensure that before proceeding with the study that all research team members have reviewed the North-West University COVID-19 Occupational Health and Safety Standard Operating Procedure as well as that of the Unit for Environmental Sciences and Management regarding COVID-19 precautions during field work.

Special in process conditions of the research for approval (if applicable):

a. Please provide the NWU-HREC with a copy of the goodwill permission letter from the Office on the Rights of the Child, granting access to the facilities to be used.

b. Please provide the NWU-HREC with copies of the permission letters from the remaining ward councilors to be included in the project, indicating that the study can proceed in the remaining wards.

c. Please provide the NWU-HREC with copies of the permission letters from the indunas to be approached in the study, indicating that the study can proceed.

As the study progresses the aforementioned conditions should be submitted to Ethics-HRECProcess@nwu.ac.za with a cover letter with a specific subject title indicating "Outstanding documents for approval: NWU-XXXXX-XX-XX." The letter should include the title of the approved study, the names of the researchers involved, that the documents are being submitted as part of the conditions of the approval set by the NWU-HREC, the nature of the document i.e. which condition is being fulfilled and any further explanation to clarify the submission.

The *e-mail*, to which you attach the documents that you send, should have a *specific subject line* indicating the nature of the submission e.g. "Outstanding documents for approval: NWU-XXXXX-XX-XX". The e-mail should indicate the nature of the document being sent. This submission will be handled via the expedited process.

The NWU-HREC would like to remain at your service and wishes you well with your study. Please do not hesitate to contact the NWU-HREC for any further enquiries or requests for assistance.

Yours sincerely,

Chairperson NWU-HREC

Current details:(23239522) G:\My Drive\9. Research and Postgraduate Education\9.1.5.4 Templates\9.1.5.4.2_NWU-HREC_EAL.docm
20 August 2019
File Reference: 9.1.5.4.2
