# Supplementary material for: ‘Through the drawings…they are able to tell you straight’: Using arts-based methods in violence research in South Africa
Source: PLOS Glob Public Health. 2023 Oct 9;3(10):e0002209. doi: 10.1371/journal.pgph.0002209 (PMC10561840; doi:10.1371/journal.pgph.0002209)
Supplement: S4 File — (DOCX) [file pgph.0002209.s004.docx]

STROBE Statement—checklist of items that should be included in reports of observational studies

|  | Item No. | Recommendation | Page  No. | Relevant text from manuscript |
| --- | --- | --- | --- | --- |
| **Title and abstract** | 1 | (*a*) Indicate the study’s design with a commonly used term in the title or the abstract | 2 | A pilot study using a cross sectional sample was conducted in rural South Africa to determine the feasibility and acceptability of using arts-based methods in research with adults and children, in preparation for a longitudinal multigenerational cohort study on mechanisms that underly the intergenerational transmission of violence. |
|  |  | (*b*) Provide in the abstract an informative and balanced summary of what was done and what was found | 2 |  |
| Introduction | | | |  |
| Background/rationale | 2 | Explain the scientific background and rationale for the investigation being reported | 3-7 |  |
| Objectives | 3 | State specific objectives, including any prespecified hypotheses | 7 | Due to the existing gaps in the research on the acceptability and feasibility of art-based methods specifically within the South African context and with victims and/or perpetrators of interpersonal violence, a pilot study was conducted. The purpose of the pilot study was firstly, to prepare for a longitudinal multigenerational cohort study on mechanisms that underly the intergenerational transmission of violence, and secondly, to observe the potential of using four arts-based research methods. This study reports on the experiences of interviewers and participants regarding the feasibility and acceptability of using these methods in violence research with adults and children. |
| Methods | | | |  |
| Study design | 4 | Present key elements of study design early in the paper | 9-12 |  |
| Setting | 5 | Describe the setting, locations, and relevant dates, including periods of recruitment, exposure, follow-up, and data collection | 7 | A pilot study was undertaken in rural Mpumalanga in South Africa (July–October 2021) (28). |
| Participants | 6 | (*a*) *Cohort study*—Give the eligibility criteria, and the sources and methods of selection of participants. Describe methods of follow-up  *Case-control study*—Give the eligibility criteria, and the sources and methods of case ascertainment and control selection. Give the rationale for the choice of cases and controls  *Cross-sectional study*—Give the eligibility criteria, and the sources and methods of selection of participants | 7 | The study comprised a convenience cross sectional sample drawn from residents in the area of young adults aged 22-30 years, children aged 4-7 years, and former caregivers of the young adults. |
|  |  | (*b*) *Cohort study*—For matched studies, give matching criteria and number of exposed and unexposed  *Case-control study*—For matched studies, give matching criteria and the number of controls per case |  |  |
| Variables | 7 | Clearly define all outcomes, exposures, predictors, potential confounders, and effect modifiers. Give diagnostic criteria, if applicable |  |  |
| Data sources/ measurement | 8* | For each variable of interest, give sources of data and details of methods of assessment (measurement). Describe comparability of assessment methods if there is more than one group | 14 | A portion of qualitative interviews were audio recorded and transcribed (with children, n=15 and young adults, n=19). In addition, three focus group discussions (FGDs) were undertaken with interviewers following pilot study completion to understand implementation and reflect on interviewer perceptions, experiences and lessons learned. The FGD guides were developed by the investigative team (five members) and comprised an intensive, team-based approach that helped strengthen insight and validity of findings. FGDs involved all 6 interviewers over a period of 4 months and included written consent. These discussions were guided in person by the project manager, who is a qualitative researcher, and were supported online in real time by the investigative team. Supplementary questions were asked by investigators during the discussions that took roughly 2 hours, were audio recorded and transcribed. |
| Bias | 9 | Describe any efforts to address potential sources of bias |  |  |
| Study size | 10 | Explain how the study size was arrived at |  |  |

Continued on next page

| Quantitative variables | 11 | Explain how quantitative variables were handled in the analyses. If applicable, describe which groupings were chosen and why |  |  |
| --- | --- | --- | --- | --- |
| Statistical methods | 12 | (*a*) Describe all statistical methods, including those used to control for confounding |  |  |
|  |  | (*b*) Describe any methods used to examine subgroups and interactions |  |  |
|  |  | (*c*) Explain how missing data were addressed |  |  |
|  |  | (*d*) *Cohort study*—If applicable, explain how loss to follow-up was addressed  *Case-control study*—If applicable, explain how matching of cases and controls was addressed  *Cross-sectional study*—If applicable, describe analytical methods taking account of sampling strategy |  |  |
|  |  | (*e*) Describe any sensitivity analyses |  |  |
| Results | | | | |
| Participants | 13* | (a) Report numbers of individuals at each stage of study—eg numbers potentially eligible, examined for eligibility, confirmed eligible, included in the study, completing follow-up, and analysed |  |  |
|  |  | (b) Give reasons for non-participation at each stage |  |  |
|  |  | (c) Consider use of a flow diagram |  |  |
| Descriptive data | 14* | (a) Give characteristics of study participants (eg demographic, clinical, social) and information on exposures and potential confounders |  |  |
|  |  | (b) Indicate number of participants with missing data for each variable of interest |  |  |
|  |  | (c) *Cohort study*—Summarise follow-up time (eg, average and total amount) |  |  |
| Outcome data | 15* | *Cohort study*—Report numbers of outcome events or summary measures over time |  |  |
|  |  | *Case-control study—*Report numbers in each exposure category, or summary measures of exposure |  |  |
|  |  | *Cross-sectional study—*Report numbers of outcome events or summary measures |  |  |
| Main results | 16 | (*a*) Give unadjusted estimates and, if applicable, confounder-adjusted estimates and their precision (eg, 95% confidence interval). Make clear which confounders were adjusted for and why they were included |  |  |
|  |  | (*b*) Report category boundaries when continuous variables were categorized |  |  |
|  |  | (*c*) If relevant, consider translating estimates of relative risk into absolute risk for a meaningful time period |  |  |

Continued on next page

| Other analyses | 17 | Report other analyses done—eg analyses of subgroups and interactions, and sensitivity analyses | 14-15 | First, four researchers reviewed and assessed at least three transcripts each of interviewer-led qualitative interviews, to appraise pilot processes and application, giving feedback to interviewers on utilization of methods, interaction with participants and quality of data gathered. Researchers discussed reflections of the transcripts reviewed (that were grouped into themes by the first author) and together with observations from the field and team meeting discussion over a 5 month period, triangulation primed inclusive discussion guides for the FGDs that followed (49). Deliberations on central findings after every FGD was concluded with the broader investigative team ensured nuanced questions were incorporated in the next round of discussions, guaranteeing all areas of investigation were being grasped and studied. A rapid analytic approach (50,51) was used to analyse data. This swift and iterative approach to data collection and analysis was undertaken to understand the pilot study, including the acceptability, feasibility and appropriateness of the study methods and materials, and chosen for its capacity to produce targeted research in a timely way to inform the main study tools and implementation that was forthcoming (52). Study investigators who analysed the data were embedded in the study with operational understanding of the context and methods, offering insight to the complexity of the study implementation. The analysis was minimally interpretive with the first author establishing an inventory of the data contents or main domains, derived from the interview and discussion guides, condensing and consolidating the data into summaries (53). Three researchers read all FGD transcripts, underscoring significant quotes that matched the inventory generated, and clarified or confirmed summarized significant findings collectively. |
| --- | --- | --- | --- | --- |
| Discussion | | | | |
| Key results | 18 | Summarise key results with reference to study objectives | 22 | Our pilot study found that using arts and play-based methods in multigenerational violence research is feasible and acceptable to participants and interviewers. These methods worked well for nearly all participants regardless of age or ability. They can enhance what researchers (and participants) can discover through their capacity to facilitate disclosure, particularly around stigmatizing and sensitive experiences like violence (16). Perhaps this is due to the increased safety these methods provide in their ability to facilitate expression and allow participants personal distance from the content of violence they are sharing with interviewers, enhancing critical thinking (54) Our study appeared to support other local research with vulnerable violence exposed participants and their perceived benefit of inclusion, having an opportunity to share adverse experiences with others in a ‘safe space’ (55) |
| Limitations | 19 | Discuss limitations of the study, taking into account sources of potential bias or imprecision. Discuss both direction and magnitude of any potential bias | 29 | Rapid data analysis helped identify the context specific issues that needed to be addressed and understood (both on the part of participants but also interviewers) related to the data collection tools utilized for the pilot. However, rapid research designs tend to use small sample sizes which complicates generalizability of findings. Also, this analysis strategy did not benefit from a deeper thematic analysis of data.  Although interviewers where trained and mentored in the methods used, there was a limitation in deepening interviews and facilitating more probing around significant participant responses. As a result, the main study employed more experienced qualitative researchers. Participants may also have been biased through social desirability. |
| Interpretation | 20 | Give a cautious overall interpretation of results considering objectives, limitations, multiplicity of analyses, results from similar studies, and other relevant evidence | 22-29 |  |
| Generalisability | 21 | Discuss the generalisability (external validity) of the study results |  |  |
| Other information | |  | | |
| Funding | 22 | Give the source of funding and the role of the funders for the present study and, if applicable, for the original study on which the present article is based |  | *Funding This study is funded by the European Research Council (ERC) under the European Union’s Horizon 2020 research and innovation programme (Grant Agreement Number 852787) and the UK*  *Research and Innovation Global Challenges Research Fund (ES/S008101/1).* |

*Give information separately for cases and controls in case-control studies and, if applicable, for exposed and unexposed groups in cohort and cross-sectional studies.

**Note:** An Explanation and Elaboration article discusses each checklist item and gives methodological background and published examples of transparent reporting. The STROBE checklist is best used in conjunction with this article (freely available on the Web sites of PLoS Medicine at http://www.plosmedicine.org/, Annals of Internal Medicine at http://www.annals.org/, and Epidemiology at http://www.epidem.com/). Information on the STROBE Initiative is available at www.strobe-statement.org.
