# Supplementary material for: ‘Through the drawings…they are able to tell you straight’: Using arts-based methods in violence research in South Africa
Source: PLOS Glob Public Health. 2023 Oct 9;3(10):e0002209. doi: 10.1371/journal.pgph.0002209 (PMC10561840; doi:10.1371/journal.pgph.0002209)
Supplement: Attachment — Submitted filename: PLoS Global Public Health reviewer response_Jun2023.docx [file pgph.0002209.s006.docx]

1. **Editor:** Please address the comments/critiques raised during peer review. In the introduction, it is important to include one paragraph describing the context of domestic violence in South Africa (prevalence, major trends, etc.).

*Thank you for highlighting this omission. The following paragraph has been added to the introduction (page 3) to contextualise the paper:*

Interpersonal violence is pervasive in South Africa and is the second leading cause of loss of disability-adjusted life years (6). Intimate partner violence is reported by 32% of women in population-based studies (7) and rates of femicide are six times the global average (6). Violence against children and adolescents is equally rife with national estimates of sexual abuse against girls at 15% and boys at 10% (8). Given these rates of violence, it is prudent to explore experiences of those exposed to better understand the mechanisms of violence and its transmission across generations (9).

2. In your discussion section, please compare your results with similar studies addressing domestic violence in SA and/or studies that utilized arts to understand better the impact of domestic violence on people’s lives.

*We have added this paragraph to the discussion section:*

In our review of the literature, we could not find studies from South Africa that investigated the use of arts-based methods utilized by fieldworkers/interviewers conducting violence research, i.e. not utilized as an intervention per se and not applied by specialist therapists. This indicates an opportunity to explore in future research to assess the utility of these methods in cross sectional studies on violence. (Page 34).

3. **Journal Requirements:** Please include a complete copy of PLOS’ questionnaire on inclusivity in global research in your revised manuscript. Our policy for research in this area aims to improve transparency in the reporting of research performed outside of researchers’ own country or community. The policy applies to researchers who have travelled to a different country to conduct research, research with Indigenous populations or their lands, and research on cultural artefacts. The questionnaire can also be requested at the journal’s discretion for any other submissions, even if these conditions are not met.  Please find more information on the policy and a link to download a blank copy of the questionnaire here: <https://journals.plos.org/globalpublichealth/s/best-practices-in-research-reporting>. Please upload a completed version of your questionnaire as Supporting Information when you resubmit your manuscript.

*This has been included. In addition, this sentence has been added to the methods section:*

Additional information regarding the ethical, cultural, and scientific considerations specific to inclusivity in global research is included in the Supporting Information (SX Checklist).

4. Please send a completed 'Competing Interests' statement, including any COIs declared by your co-authors. If you have no competing interests to declare, please state "The authors have declared that no competing interests exist". Otherwise please declare all competing interests beginning with the statement "I have read the journal's policy and the authors of this manuscript have the following competing interests:"

*This has been done.*

5. We noticed that you used "unpublished data" in the manuscript. We do not allow these references, as the PLOS data access policy requires that all data be either published with the manuscript or made available in a publicly accessible database. Please amend the supplementary material to include the referenced data or remove the references.

*This has been done.*

6. Please review your reference list to ensure that it is complete and correct. If you have cited papers that have been retracted, please include the rationale for doing so in the manuscript text, or remove these references and replace them with relevant current references. Any changes to the reference list should be mentioned in the rebuttal letter that accompanies your revised manuscript. If you need to cite a retracted article, indicate the article’s retracted status in the References list and also include a citation and full reference for the retraction notice.

*Please note references have been checked and are complete and correct.*

7. **Reviewer #1**: This is a very interesting study on using art based methods for collection of qualitative data on violence. The authors have ventured to understand the feasibility and experiences of the interviewers and the acceptability of this method in engaging participants to collect their information on experience of violence. What was most impressive about the paper was the strong grounding in a theoretical explanation of art based data collection methods. The authors have done a great job of explain the details of the methods they used for collecting data. They have explained the efforts in training of the interviewers and sensitivity and reflexivity of the interviewers. The details in which the methods are explained helps the reader understand the scientific rigour in the research. The findings are explained well with appropriate quotes. The discussion is elaborate and appropriate and well drawn out. I have some minor comments for improvement:

*Many thanks Reviewer 1 for your feedback.*

8. The main aim seems to be to understand feasibility and acceptability of art based interview methods in qualitative studies of violence. The results focus on the experiences of conducting these art based interviews. However, I would like to see some data specifically on feasibility - was the method more engaging? was the method easier than others? did the method involve less expenditure? was it more time efficient? what aspects of feasibility were analysed? I would also like to see some data on acceptability? were the participants more actively engaged and did they accept the method. Was there any instance where participants could not draw? was there reluctance to draw? such findings will enhance the understanding of the experience of using art based methods.

*Thank you for this comment. We have included additional information in the findings section to discuss feasibility and acceptability more intentionally:*

There were no refusals in using the methods and all participants were able to complete a drawing. The process of drawing was more important than the perceived value of the end product and all could participate successfully, regardless of drawing ability. For child participants in particular, drawings were easily completed and play methods experienced as fun. However, especially with older caregivers, who might have been illiterate, there was a sense of fear in not being able to write or draw and more sensitivity and encouragement was required on the part of the interviewer. In these situations, interviewers typically modelled how to draw and gave the participant the opportunity to try in a non-judgemental encounter. (Page 17).

The ability to reflect on relationships in childhood and in present family constellations through the KFDs across time in their lives may have been particularly helpful in recognising intergenerational patterns of behaviour and interpersonal relationship styles that transmit over time. (Page 19).

Interviewers revealed the powerful potential of using these ways of working with participants; a realisation that came from their experiential use of the methods from training. It also highlighted the need for these to be used with sensitivity and care. (Page 21).

We did find that some participants, typically those less developmentally advanced, struggled to engage, which could be the result of behaviours not being managed well in the interview or being easily emotionally triggered by the tasks required. (Page 24).

This ‘difficult behaviour’ [of some child participants] was pronounced in the house plan activity with the squiggle drawing and KFD typically being easier to complete independently. (Page 24).

Thinking about what the image may reflect or mirror back (86) may need to be facilitated and supported by the interviewer within the triangular relationship and may be too sophisticated a skill to master in some circumstances. (Page 29).

Although interviewers where trained and mentored in the methods used, there was a limitation in deepening interviews and facilitating more probing around significant participant responses. As a result, the main study employed more experienced qualitative researchers. (Page 34).

9. What measures were adopted to ensure credibility, transferability, dependability and confirmability of the qualitative findings? was some kind of triangulation of the findings possible? was it attempted?

*Thank you for raising this – there were other measures adopted to triangulate findings that were not initially included in the text but have now been highlighted more intentionally as follows:*

A portion of qualitative interviews were audio recorded and transcribed (with children, n=15 and young adults, n=19). Interviewers completed fieldnotes after these to assess distress and feasibility of methods used. In addition, three focus group discussions (FGDs) were undertaken with interviewers following pilot study completion to understand implementation and reflect on interviewer perceptions, experiences and lessons learned. The FGD guides were developed by the investigative team (five members) and comprised an intensive, team-based approach that helped strengthen insight and validity of findings. FGDs involved all 6 interviewers over a period of 4 months. These discussions were guided in person by the project manager, who is a qualitative researcher, and were supported online in real time by the investigative team. Supplementary questions were asked by investigators during the discussions that took roughly 2 hours, were audio recorded and transcribed. Observation notes were undertaken when investigators were in the field and the investigative team met weekly to discuss implementation, with meeting notes recorded, and reflect on data jointly as it was gathered and submitted. (Under Data Collection on page 15).

*In addition, this text is on page 16 under Data Analysis:*

Researchers discussed reflections of the transcripts reviewed (that were grouped into themes by the first author) and together with observations from the field and team meeting discussion over a 5 month period, triangulation primed inclusive discussion guides for the FGDs that followed (55) as well as themes that were identified in participant transcripts.

10. How were the participants in the violence research sampled, how were the interviewers sampled for this study? was there some purposiveness in the sampling?

*Participants were conveniently sampled from the area our main study participants live in for our pilot (our main study follows participants that have already engaged in this research previously).*

A pilot study was undertaken in rural Mpumalanga in South Africa (July–October 2021) (34). The study comprised a convenience cross sectional sample drawn from residents in the area of young adults aged 22-30 years, children aged 4-7 years, and former caregivers of the young adults. (Page 8).

*Interviewers lived in the same areas participants lived in but were recruited for their role as interviewers in this study through regular advertising and hiring mechanisms. They were not purposively recruited but the job was advertised in the area of the study. We have added the following to explain this more fully in the text:*

Interviewers were recruited through formal hiring processes but came from the same communities participants lived in, with language ability and insight into community that was predicted to lead to high-quality data collection. (Page 8).

11. **Reviewer #2**: Thank you for this interesting paper which I enjoyed reading.

*Thank you Reviewer 2 for your interest in our paper.*

12. You provide a large number of references in support of the points you make - can you include in the reporting on what each of the various studies says, where the study was done/what the population was that the author(s) have engaged with? As you note yourself the context can influence how methods are used - so it would be helpful to provide the reader with the information on where each study was done. Is reference 64 a paper from the results of this pilot? If so, please say so, given this is a paper about methods it would be helpful for the reader to know where the study findings can be found.

*We have added text to substantiate some points we make as follows:*

There is burgeoning evidence of the effectiveness of their use in South African health research; Gittings and colleagues (2022) highlight their value in reflecting on and engaging with sensitive topics such as HIV with an adolescent community advisory group over a twelve year period (4), and Honikman and colleagues (2020) support their effectiveness in facilitating dialogue with nurses in primary healthcare settings in an attempt to integrate mental health services more compassionately with patients; but less is known about their utility in violence-related research in a low-resource setting like South Africa. (Page 3)

*Reference #64 is not a paper from the pilot study detailed here.*

13. In the first paragraph you mention the utility of the method for violence-related research - but you don't actually give any context for why you are interested in this. Can you provide a sentence background as to why this was your research area?

*Certainly – we have added the following to the first paragraph:*

We were interested in exploring their use in preparation for a large, longitudinal cohort study investigating multigenerational transmission of violence in South Africa.

14. Line 92 - should the term `emotionally loaded events' be in quotes since it seems to be taken from reference 17.

*We have added quotes to this phrase.*

15. Line 99 following you include a very useful piece on the interviewer experience - here you mention the novelty of papers and pencils being available in low resource settings. But this is not just a matter for the interviewers to manage - it also affects the respondent's experience. We know nothing about the socio-economic profile of the participants - can you say more about their background, the place, and the type of education they will have had - and how well that may have prepared them for research using novel methods?

*We agree and have offered more contextual information in the introduction:*

…They may also offer a platform that is more easily engaged with where literacy levels may be low, as is the case in South Africa. In the most recent Progress in International Reading Literacy Study (PIRLS), 81% of Grade 4 children (10 year olds) are unable to read for meaning, a statistic that hasn’t changed much in the last decade (13). (Page 4)

… The South African education system has been described as ‘essentially dysfunctional’ where resources are scarce in the public sector (26). (Page 5)

16. Line 211 - what are quantifiable qualitative data? Information contained in a narrative that you count? I found this information on the quantitative interview rather confusing - perhaps you could insert a box showing a sequence of methods used in the interview?

*We are in agreement that this phrasing is confusing and have omitted the word ‘quantifiable’ from the text.*

17. Line 279ff -- did adults and children provide written consent and assent for the interviews? You only mention written consent for the FGDs.

*Thank you for noting this omission – yes, parents provided written consent for themselves and their children before they began interviews. The text has been altered to reflect this:*

Parents gave written informed consent for their participation and written informed consent for their children’s participation. Parents were interviewed before children; then children were invited to participate and assented to the research.

18. The results section would benefit from some substantial reworking. You are over reliant on quotes at the moment and depend on the reader following the quotes and being able to draw from each of the quotes the meaning you wish to highlight. Please help the reader more - shift the balance so you paraphrase/ provide interpretative text much more, so the balance is shifted to have short, key quotes with the bulk of the text being your own words, to carry the reader through your arguments. The discussion is very long - and some of the material in that may work better in the results section, as you offer explanatory text around what an interviewee or the interviewer says about the experience of the methods.

*The findings and discussion sections have been substantially altered. A bulk of text from the discussion has been reworked into the findings and the findings bolstered to guide the reader more thoughtfully through the section. We trust it is more meaningful and easy to read in this current iteration.*

19. The reference list requires some attention - there are some references with titles wholly in upper case, for example.

*Thank you for bringing that to our attention. The references have been reworked.*
